# Flow Cytometric Immunobead Assay for Detection of BCR-ABL1 Fusion Proteins in Chronic Myleoid Leukemia: Comparison with FISH and PCR Techniques

**DOI:** 10.1371/journal.pone.0130360

**Published:** 2015-06-25

**Authors:** Anna Grazia Recchia, Nadia Caruso, Sabrina Bossio, Mariavaleria Pellicanò, Laura De Stefano, Stefania Franzese, Angela Palummo, Vincenzo Abbadessa, Eugenio Lucia, Massimo Gentile, Ernesto Vigna, Clementina Caracciolo, Antolino Agostino, Sara Galimberti, Luciano Levato, Fabio Stagno, Stefano Molica, Bruno Martino, Paolo Vigneri, Francesco Di Raimondo, Fortunato Morabito

**Affiliations:** 1 Biotechnology Research Unit, ASP Cosenza, Aprigliano, Italy; 2 Hematology Unit, Azienda Ospedaliera Annunziata di Cosenza, Cosenza, Italy; 3 Department of Oncology, Hematology and Bone Marrow Transplantation Unit, University of Palermo, Policlinico P. Giaccone, Palermo, Italy; 4 Centro Trasfusionale Ospedale, Azienda Sanitaria Provinciale 7, Ragusa, Italy; 5 Department of Clinical and Experimental Medicine, University of Pisa, Pisa, Italy; 6 Medical Oncology Unit, Hematology-Oncology Department, Azienda Ospedaliera Pugliese-Ciaccio, Catanzaro, Italy; 7 Divisione di Ematologia, Ospedale Ferrarotto, Università degli Studi di Catania, Catania, Italy; 8 U.O.C. di Ematologia dell'Azienda"Bianchi-Melacrino-Morelli" di Reggio Calabria, Reggio Calabria, Italy; 9 Dipartimento di Scienze Mediche e Pediatriche, Università degli Studi di Catania, Catania, Italy; B.C. Cancer Agency, CANADA

## Abstract

Chronic Myeloid Leukemia (CML) is characterized by a balanced translocation juxtaposing the Abelson (ABL) and breakpoint cluster region (BCR) genes. The resulting BCR-ABL1 oncogene leads to increased proliferation and survival of leukemic cells. Successful treatment of CML has been accompanied by steady improvements in our capacity to accurately and sensitively monitor therapy response. Currently, measurement of BCR-ABL1 mRNA transcript levels by real-time quantitative PCR (RQ-PCR) defines critical response endpoints. An antibody-based technique for BCR-ABL1 protein recognition could be an attractive alternative to RQ-PCR. To date, there have been no studies evaluating whether flow-cytometry based assays could be of clinical utility in evaluating residual disease in CML patients. Here we describe a flow-cytometry assay that detects the presence of BCR-ABL1 fusion proteins in CML lysates to determine the applicability, reliability, and specificity of this method for both diagnosis and monitoring of CML patients for initial response to therapy. We show that: i) CML can be properly diagnosed at onset, (ii) follow-up assessments show detectable fusion protein (i.e. relative mean fluorescent intensity, rMFI%>1) when BCR-ABL1^IS^ transcripts are between 1–10%, and (iii) rMFI% levels predict CCyR as defined by FISH analysis. Overall, the FCBA assay is a rapid technique, fully translatable to the routine management of CML patients.

## Introduction

Chronic Myeloid Leukemia (CML) is characterized by a balanced translocation, fusing the Abelson oncogene (ABL1) on chromosome 9q34 with the breakpoint cluster region (BCR) on chromosome 22q11.2, t(9;22)(q34;q11.2), more commonly known as the Philadelphia chromosome. The molecular product of this translocation is the BCR-ABL1 fusion oncogene. Detection of t(9;22) is generally carried out at the chromosome level using karyotyping or fluorescence *in situ* hybridization (FISH) or by real-time quantitative PCR (RQ-PCR) at the mRNA level. The availability of these techniques is generally restricted to specialized laboratories in reference centers with well-trained personnel. Furthermore, these techniques are time consuming and dependent on laboratory-specific workloads: usually requiring on average 1–2 days for FISH and PCR techniques, or 1–2 weeks for karyotyping analysis.

CML treatment has received considerable attention since the introduction of imatinib mesylate (IM), the first tyrosine kinase inhibitor (TKI) directed specifically against BCR-ABL1 catalytic activity. Today, IM represents one of the established front-line therapies for CML together with second-generation TKIs [1–5]. While these drugs have profoundly modified the natural history of CML [6–7], they have also generated a need for frequent molecular monitoring that has become mandatory for all patients [8]. In turn, this has created a challenge for the workload of CML laboratories, considering the huge increase in the prevalence of the disease [9, 10]. CML monitoring presently relies on i) bone marrow cytogenetics in the first 12 to 18 months; ii) continuous measurement of BCR-ABL1 transcripts by RQ-PCR assays, and iii) testing for BCR-ABL1 tyrosine kinase domain (TKD) mutations in selected cases. Weerkamp et al [11] developed a flow cytometric immunobead assay (FCBA) for the detection of BCR-ABL1 fusion proteins in cell lysates, using a bead-bound anti-BCR catching antibody and a fluorochrome-conjugated anti-ABL detection antibody intended for the rapid diagnosis of Philadelphia positive acute lymphoblastic leukemia (ALL). Testing of 145 CML patient samples showed full concordance between the FCBA and RQ-PCR of fusion gene transcripts [11], demonstrating that the FCBA detects all BCR-ABL1 proteins in leukemic cells with high specificity and sensitivity [11]. However, to date, there are no studies evaluating whether the FCBA could be of clinical utility in evaluating MRD in CML patients.

The aims of the present study include: i) the application of the FCBA in a clinical laboratory setting for the rapid diagnosis of CML in order to evaluate specificity and selectivity of BCR-ABL1 detection in comparison with routine RQ-PCR testing; ii) evaluation of the potential use of FCBA in the follow-up of patients treated with TKIs and monitored in parallel with routine RQ-PCR testing; and iii) comparison of the sensitivity and specificity of FCBA and FISH in the clinical setting.

## Materials and Methods

### Sample Collection

Freshly collected peripheral blood (PB) and bone marrow (BM) samples were obtained from patients visiting outpatient clinics from centers participating in the Sicily and Calabria CML REgional ENterprise (SCREEN) network in accordance with the Declaration of Helsinki. Samples were centralized at the Cosenza laboratory where they were processed by lysis of erythrocytes to obtain total WBC. The project was approved by the local Ethics Committee of the Azienda Policlinico Vittorio Emanuele of Catania for the secondary use of remaining diagnostic material isolated from patients suspected to have hematological malignancies. Informed consent was obtained orally from the participants and recorded in clinical files; the data were analyzed anonymously.

Presence of the BCR-ABL1 protein was investigated on a total of 278 PB and BM samples belonging to 122 patients (S1 Table). Of these, 153 serial CML follow-up samples (including n = 39 matched BM and PB) were tested in patients after 3 months of diagnosis up to a negative result in the FCBA protein assay (S1 Table). Moreover, a subset of 55 CML patients was tested both at diagnosis and follow-up. All patients were referred to participating Institutions between February 2011 and November 2013.

### Healthy Controls

Peripheral blood samples were obtained from healthy donors after receiving informed consent. Total white blood cells (WBC) were isolated as described above.

### Flow Cytometry Analysis of the BCR-ABL1 Protein

For the identification of the BCR-ABL1 fusion protein, we employed the BCR-ABL1 Protein Kit (FCBA, BD Biosciences). The FCBA consists of an immunoassay that qualitatively identifies the presence of the BCR-ABL1 protein in the lysates of the leukemic cell population. After lysis of the leukemic cells, the fusion proteins are released and are then recognized by an anti-BCR antibody coupled to a bead and a (Phycoerythin, [PE])-labeled anti-ABL antibody [11]. Lysates from normal peripheral leukocytes (WBC) and from the BCR-ABL1^+^ K562 cell line (see below) were used as negative and positive controls, respectively. Tests were performed according to the manufacturer’s instructions with few modifications. Specifically, whole blood/marrow specimens containing 30×10^6^ WBCs cells were incubated with 50 ml of PharmLyse lysing buffer (BD Biosciences) for ten minutes at room temperature with occasional mixing to lyse red blood cells. Cells were then washed twice by adding PBS with 5% FBS and counted using an automated cell counter. Cells were then diluted to a concentration of 3x10^6^ cells and pelleted for the following steps. A volume of 250 μL of Pretreatment Buffer obtained by diluting the 1X stock of BCR-ABL1 Pretreatment A (BD Biosciences) and the 1X stock of BCR-ABL1 Pretreatment B (BD Biosciences) were added to both samples and controls; samples were then incubated on ice for ten minutes and washed once by adding PBS with 5% FBS. Thereafter, samples and controls were incubated for 15 minutes with 100 μL of the 1X stock of the BD Lysate Treatment Reagent (BD Biosciences) and then centrifuged at 20,000×g for ten minutes at 4°C. Fifty μL of the cell lysates, from both samples and controls, were incubated for two hours in the dark with 50 μL of an anti-BCR antibody coupled to a bead (BD Biosciences) and 50 μL of the PE-labeled anti-ABL antibody (BD Biosciences). After washing with CBA Wash Buffer (BD Biosciences Pharmigen), the bead-pellet samples were re-suspended in 300 μL of the CBA Wash Buffer and acquired using a dual-laser flow-cytometer. The BD FACSCanto II flow-cytometer running BD FACSDiva software version 6.0, capable of detecting forward and side light scatter and fluorescence emissions at both 578 nm and either 660 or 800 nm (or both) was set using the Cytometer Setup and Tracking (CS&T) Bead system according to the manufacturer’s guidelines (BD Biosciences).

Absence of the BCR-ABL1 fusion protein (negative cut-off value [Normal cut-off]) was defined utilizing the mean + 2 standard deviations (SD) of PE MFI of at least 20 samples of normal peripheral leukocytes for each kit. Re-calculation of negative cut-off values was performed for each new kit used as recommended by the Manufacturer. Standardization of Mean Fluorescence Intensity (MFI) values using mean Normal cut-off values was necessary for comparative analysis across kits; therefore a relative MFI% value (rMFI%) was calculated as follows [(MFI_Sample_−MFI_Normal Cut-off_) / MFI_Normal cut-off_] × 100; where a rMFI% value >1 was scored as a positive test.

### Specificity and Sensitivity Testing of the BCR-ABL Protein Kit with Cell Lines

The specificity of the FCBA kit was confirmed by analyzing cell lysates from CML leukemic cell lines with different types of fusion proteins. All leukemic cell lines were purchased from the German Collection of Micro-organisms and Cell cultures (DSMZ GmbH, Braunschweig, Germany): Philadelphia chromosome-positive cell lines expressed p210 BCR-ABL1 K562 (transcript e14a2) or BV173 (transcript e13a2) and SD-1 with p190 BCR-ABL (transcript e1a2). Since we did not have a cell line expressing the atypical p230 BCR-ABL protein, we tested a patient positive for the rare p230 transcript (MA230). The HEL and HL60 cell lines were used as BCR-ABL1 negative controls.

To mimic leukemic samples with varying tumor loads, K562 cells were mixed at different concentrations with either BCR-ABL1-negative cells or normal WBCs. The sensitivity and detection limit of the BCR-ABL1 Protein Kit was therefore determined by analyzing serial dilutions of the above samples. Standardized cell concentrations and total cell numbers were determined using an automated cell counter in concentrations ranging from undiluted K562 cells (100%) to K562 concentrations as low as 0%. All analyses were carried out in duplicate. MFI values detected in 100% BCR-ABL1-negative cell lines were used as negative control values at every testing session.

### Comparison with FISH and RQ-PCR

FISH and quantitative assays were performed by clinical laboratories as part of routine clinical diagnostic procedures at disease onset and follow-up on PB and BM samples. PCR analysis of the different types of BCR-ABL1 fusion transcripts was performed with the standardized Europe Against Cancer (EAC) protocols using TaqMan based RQ-PCR [10]. The ABL internal control gene was used as control and levels of BCR-ABL1 mRNA transcript levels were reported according to the International scale (IS) [8]. FISH was performed according to standardized protocols using a Dual Color, Dual Fusion Translocation Probe Set [LSI ABL1 targeting region 9(q34) and LSI BCR targeting region 22(q11.2)] (Abbott Laboratories, Chicago, IL, USA).

### Statistical analysis

Statistical analyses were performed using the Mann-Whitney *U* test to compare mean MFI values between different BCR-ABL1 positive samples in different experimental conditions. The relation between MFI values and age of the samples was evaluated by the Spearman's rank correlation coefficient. For categorical variables, statistical comparisons (i.e. FISH analysis *versus* BCR-ABL1 flow-cytometry detection) were performed using two-way tables for the Fisher’s exact test. A value of *P* < 0.05 was considered statistically significant. The best cut-off point for BCR-ABL1^IS^ levels discriminating cases with a positive FCBA from a negative FCBA was sought by constructing receiver operating character (ROC) curves.

## Results

### BCR-ABL1 Protein FCBA Optimization Experiments

In initial optimization experiments on clinical samples, it was observed that lysing 1 to 2 mL of whole blood as indicated by the manufacturer was sufficient for detection of fusion proteins for a patient at diagnosis when WBC counts/mL in whole blood are substantially elevated in most cases in this phase (WBC range, 11.64–560.82 x 10^3^ cells/uL). Using this same sample volume for patients in follow-up, particularly those in complete hematologic remission (CHR), blood counts return to normal, and this may result in potential false negative results. In fact, according to Gabert et al guidelines [12], for MRD RQ-PCR of follow-up patients, it is necessary to begin with a sufficient pool of (10–20 million) WBCs rather than blood volumes; similarly in FISH analysis 10 million cells/mL are used in culture to obtain 200 evaluable nuclei. We suggest starting with a sample pool containing standardized cell concentration of 30 x 10^6^ WBCs obtained from whole blood cell counts in order to safeguard against losses due to centrifugation and WBC recovery. After erythrocyte lysis, and to ensure that a sufficient number of neoplastic CML cells were included for MRD testing, WBCs were counted and 3 x 10^6^ cells were used for the subsequent analysis according to the manufacturer’s recommendations.

Comparing results in fresh versus DMSO frozen lysates of positive cell lines, we observed a notable reduction in MFI even when cells were cryopreserved for only 24 hours (S1 Fig). Indeed, comparing results from fresh or DMSO frozen total leukocytes obtained from the same patients we found notable variations in PE values and in detectable BCR-ABL1 protein (S1 Fig), particularly in those samples close to the test’s negative cut-off value. Therefore, due to the observed instability of the BCR-protein, freezing and thawing samples for this BCR-ABL1 FCBA is not recommended.

We next performed a time course experiment storing patient samples at room temperature for up to 5 days. Test positivity of the same sample slowly declined even after 48 hours, which, as expected was directly correlated with cell viability (S2 Fig), and not due to loss of total protein concentration as determined by Bradford assays. Overlapping results were obtained after freeze/thaw cycles using either PB or BM cell samples from the same patient (S2 Fig). These observations suggested that it was necessary to work with fresh samples within 24 to 48h of withdrawal for best results. It is known that the BCR-ABL1 protein is particularly sensitive to degradation within primary cells, a difference that may be due to protease activity released from neutrophil granules [13]. Hence, we included a BCR-ABL1-positive sample/or cell line as a positive control for each patient sample tested, as recommended by the manufacturer to control for efficient cell lysis steps and bead detection.

### Sensitivity of the BCR-ABL1 Immunobead Assay on Cell lines and on CML Patient Samples Validated in Different Conditions

**Fig 1:**
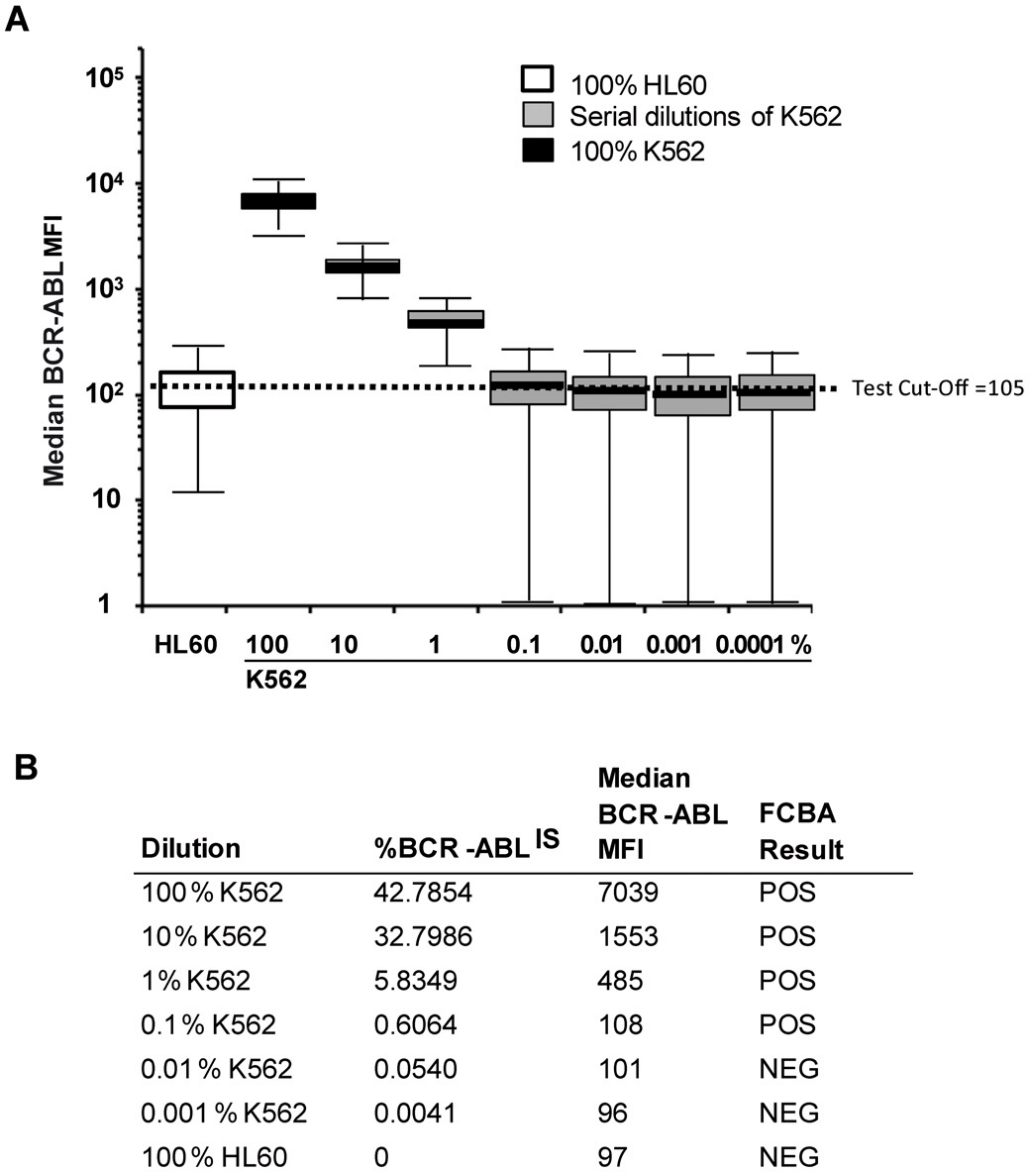
Detection limit using FCBA BCR-ABL1 protein serial dilution curve. The CML cell line K562 was diluted in the BCR-ABL-negative cell line, HL60. Negative cut-off median PE value for this series was 105. Assay sensitivity was compared with real-time quantitative PCR (RQ-PCR) of BCR-ABL1 transcripts for each dilution and standardized according to the International scale (IS). The protein assay sensitivity indicated a loss of sensitivity of the FCBA assay at a dilution between 0.1 and 0.01% K562 which corresponds to a %BCR-ABL1/ABL^IS^ mRNA ratio between 0.054 and 0.60%.

We first performed dose limiting and time course experiments using BCR-ABL1^POS^ and BCR-ABL1^NEG^ cell lines. FCBA results using BCR-ABL1^NEG^ (HEL, HL60) and BCR-ABL1^POS^ (p210: K562, BV173; p190: SD1) cell lines were concordant with the detection of oncogene transcripts by RQ-PCR independently of the breakpoint position in the BCR gene. Using serial dilutions of K562 cells in HEL cells, the detection limit of the FC assay was determined as >0.1% BCR-ABL1^IS^ (Fig 1).

**Fig 2:**
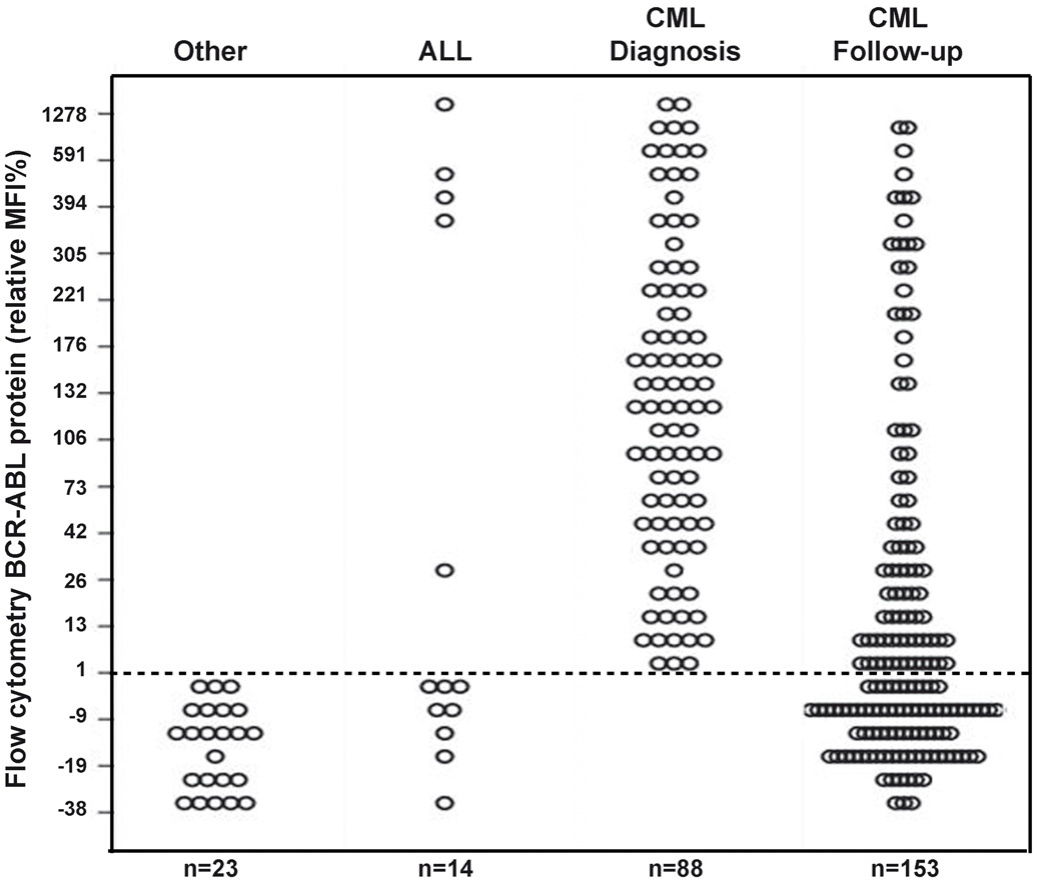
Screening clinical samples for BCR-ABL1 protein. A total of 278 cellular samples were screened for the presence of BCRABL1 protein using the FCBA assay; results are stratified by the Relative MFI% BCR-ABL. The cut-off MFI value of the Negative Control group was calculated as the mean MFI plus two standard deviations, this value was re-calculated for each test kit utilized. Relative %MFI (rMFI%) was then calculated as [(MFI_Sample_−MFI_Normal_ cut-off) / MFI_Normal_ cut-off] × 100 in order to compare data across kits. A relative MFI%>1 indicates a positive test. Twenty-three samples from other hematological disorders (including 4 AML), 14 suspected ALL and 88 samples from patients with neutrophilia and/or thrombocytosis suspected of CML diagnosis; and 155 CML serial follow-up samples were studied. See S1 Table for patient details.

The flow-cytometry BCR-ABL1 immunobead assay was tested on 278 samples (BM = 51 and PB = 74) (S1 Table). A total of 88 PB samples from normal healthy controls were used to determine test cut-off values. BCR-ABL1 protein expression was then investigated in 55 patients with CML in therapy with TKI at follow-up, for a total of 153 samples tested (Fig 2 and S1 Table). Overall, 105 samples tested negative and 173 tested positive for the BCR-ABL1 FCBA. The BCR-ABL1 protein was detected in all onset samples (88/88) and in 52.3% (80/153) of samples from follow-up patients (follow-up samples ranging from 3 months to 36 months). Of the 80 FCBA-positive samples 77 were available for RQ-PCR analysis with BCR-ABL1^IS^ transcripts ranged between 1–10%. In the remaining 73 patients with undetectable fusion protein (rMFI% ≤1), all available for RQ-PCR analysis, BCR-ABL1^IS^ levels were <1%. In addition, parallel BM and PB from the same patient were tested in 50 CML samples. The FCBA showed a strong correlation (Rho = 0.823, *P* < 0.0001) in test results indicating that either BM or PB samples could be used for the purposes of this test with satisfactory overlapping results. Of note, the 18 patients with other lymphoid or myeloid hematological disorders tested negative in the FC immunoassay also were confirmed negative by RQ-PCR (Fig 2). Only 5 of the 14 ALL samples gave a positive result by FCBA and were confirmed by RQ-PCR assays. This finding also confirms the utility of this test for a rapid screening in ALL where a quick therapeutic intervention is clearly warranted [14].

### Comparison of BCR-ABL1 Protein Assay in CML Patients with RQ-PCR

**Fig 3:**
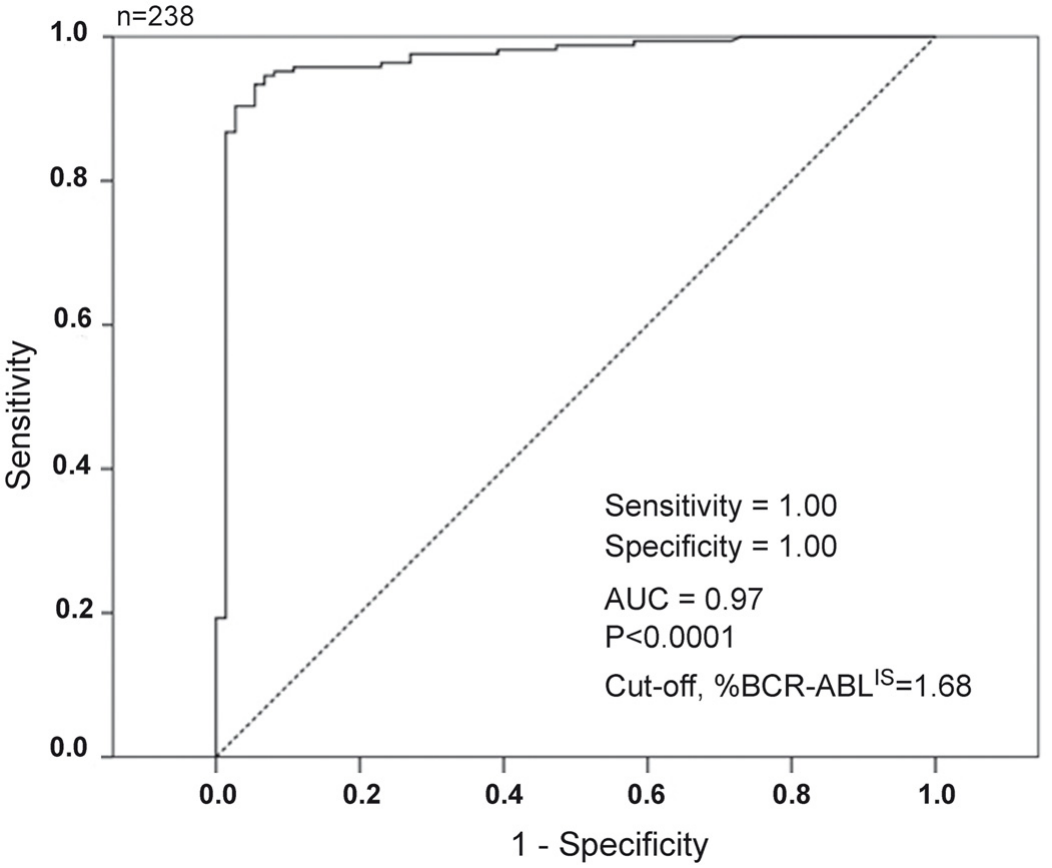
Determination of a BCR-ABL^IS^ cut-off level. Receiver operator curve (ROC) analysis was used in 238 samples to determine the best BCR-ABL1^IS^ cut-off level [1.68% (AUC = 0.97, *P*<0.001)] able to discriminate cases with a positive from negative FCBA result.

**Fig 4:**
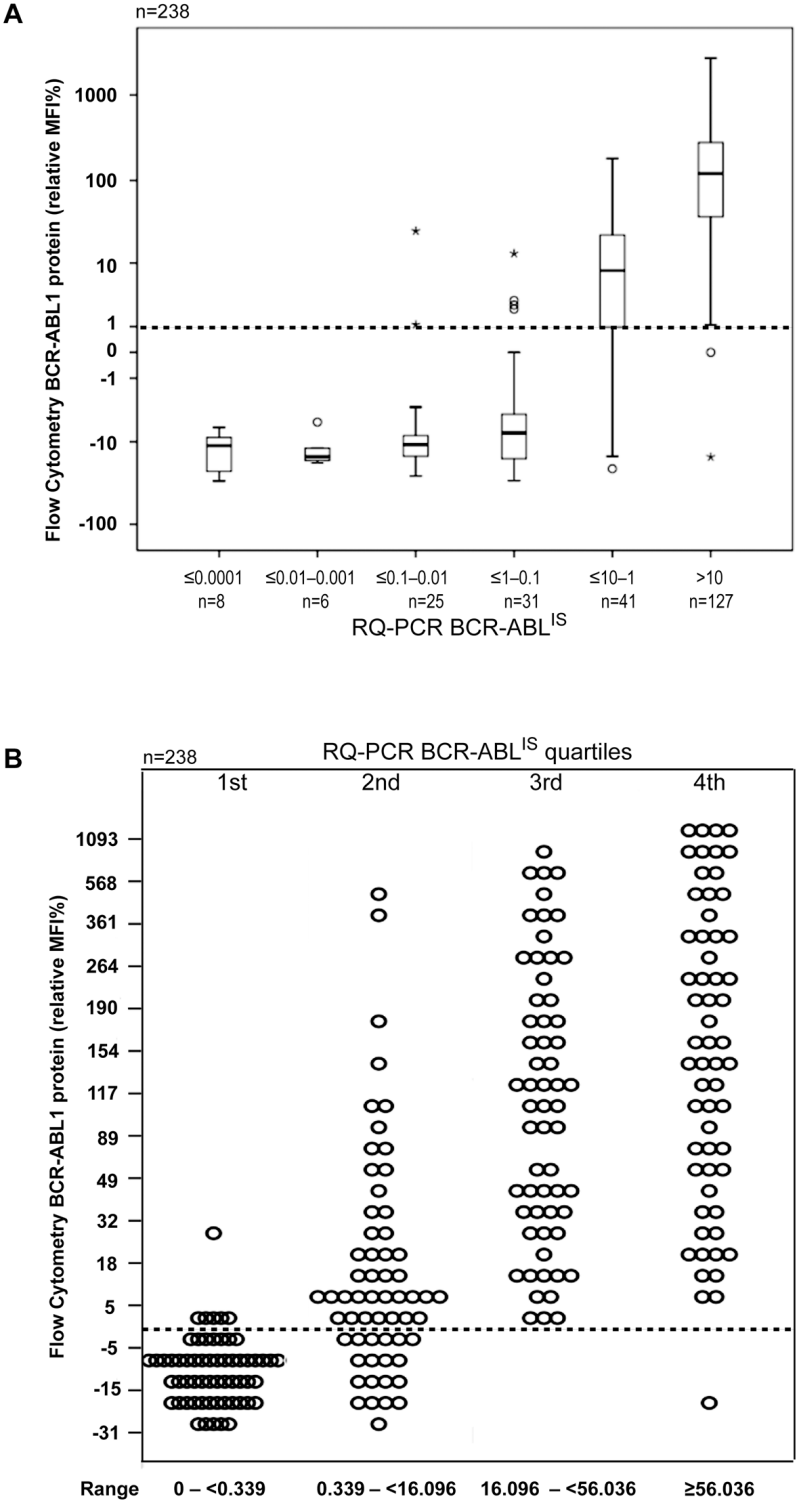
Screening Clinical Samples for BCR-ABL1 Protein. (A) A total of 238 cellular samples were screened for the presence of BCR-ABL1 protein; results are stratified by the BCR-ABL1 quantitative real-time PCR (RQ-PCR) results for the same sample. The cut-off MFI value was calculated as in Fig 2 (see Materials and Methods). (B) Dividing all the BCR-ABL1^IS^ levels into quartiles, shows a degree of linearity between BCR-ABL1 transcript and protein levels in the CML cases examined in (A).

We analyzed the specificity and sensitivity of the BCR-ABL1 FCBA Assay in primary cells by assaying for BCR-ABL1 protein in patients undergoing treatment with a TKI. Of the 241 CML samples tested in FCBA (S1 Table), 238 samples were available for RT-PCR analysis. All proteins translating the BCR-ABL1 fusion transcripts p190, p210, p190/210, including one patient who carried the p230 transcript (n = 2 samples, BM and PB, respectively), were detected by the FCBA. The fusion transcript was also confirmed by RQ-PCR in all FCBA positive patients. ROC analysis recognized 1.68% (AUC = 0.97, *P* < 0.001) as the best BCR-ABL1^IS^ cut-off level discriminating cases with a positive from negative FCBA result (Fig 3). However, 65/73 (89.0%) cases negative by FCBA proved positive by RQ-PCR (BCR-ABL1^IS^, median 0.168, range 0.001–3.76). Only 8/73 cases had undetectable transcripts (MR4) in both FCBA and RQ-PCR. Of those cases with detectable transcripts (FCBA-positive), 55/65 (84.6%) had a BCR-ABL1^IS^ <1 (range 0.001–0.771) and 10/65 (15.4%) samples had a BCR-ABL1^IS^ ≥1.0 (range 1.044–3.78) (Table 1). Overall, in patients who were in complete cytogenetic response (CCyR, defined as a BCR-ABL1 RQ-PCR <1%) [8], the detectable BCR-ABL1 protein was either extremely low or undetectable (FCBA-negative). However, in patients who failed to achieve a CCyR (i.e. those with a BCR-ABL1 ratio of 1–10%) BCR-ABL1 protein was easily detected (Fig 4A). In patients with newly diagnosed CML (BCR-ABL1 ratio >10%) BCR-ABL1 protein levels were very high (Fig 4A). Dividing all the BCR-ABL1^IS^ levels into quartiles (Fig 4B), it is clear that there is a degree of linearity between BCR-ABL1 transcript and protein levels in the CML cases examined, suggesting that the development of a standardized quantitative protein scale may represent an alternative method of rapidly evaluating disease burden in CML, albeit remaining a less sensitive technique that RQ-PCR.

**Table 1:** Comparison of Flow-Cytometric Bead Assay (FCBA) vs. RQ-PCR for Detection of BCR-ABL1 Expression in CML Follow-Up.

Molecular response	FCBA-Negative	FCBA-Positive
(According to ELN guidelines)	*n* samples (RQ-PCR range)	*n* samples (RQ-PCR range)
>10%	0	39 (10.258–195.807)
≤10–1% CHR	10 (1.044–3.769)	31 (1.198–9.914)
≤1–0.1% CCyR	26 (0.121–0.771)	5 (0.143–0.519)
≤ 0.1% MMR	37 (0–0.100)	2 (0.028–0.073)

N = 150 serial samples, belonging to 54 CML in follow-up* (*note that 1 CML patient with p230 transcripts was not included in this analysis).

Abbreviations: FCBA, Flow-cytometric Bead Assay, CHR = complete hematologic remission, CCyR = complete cytogenetic remission, MMR = major molecular response; (RQ-PCR) = quantitative real-time PCR; ELN = European Leukemia Net [8]

### Comparison of BCR-ABL1 Protein Assay and FISH

Our next aim was to determine whether the FCBA BCR-ABL1 could substitute for FISH analysis in follow-up of CML patients within the first year of TKI therapy to monitor early achievement of a cytogenetic response. Patients who are in CCyR are defined either by negative standard cytogenetic or negative FISH analysis on bone marrow or as having BCR-ABL1^IS^ RQ-PCR < 1%) [8]. We analyzed a total of 105 serial samples (belonging to 37 patients, from diagnosis and follow-up) for both FISH and BCR-ABL1 FCBA (Table 2). Of the 105 samples examined, 72/105 tested positive for both FISH and BCR-ABL1-FCBA, while 27/105 tested negative for both FISH and BCR-ABL1-FCBA. Interestingly, the BCR-ABL1-FCBA assay tested positive for 6 cases (rMFI%, range 0.97–8.7%) that had tested negative in FISH. All 6 patients were tested for MRD and showed BCR-ABL1^IS^ RQ-PCR ranging from 0.313 to 10.258. Overall these data indicate that FCBA assay is able to significantly predict FISH results (*P* < 0.0001) and therefore this flow cytometry test could theoretically replace the more time-consuming and expensive traditional FISH examination.

**Table 2:** Comparison of FISH analysis and BCR-ABL1 Flow-cytometry Bead Assay on CML samples (N = 105 serial samples in 37 patients).

*n* = 105	FISH-Negative	FISH-Positive	Totals
FCBA-Negative	27	0	27
FCBA-Positive	6*	72	78
**Totals**	33	72	105

* FCBA = Flow-cytometry Bead Assay; rMFI% range, 0.968–8.89; %BCR-ABL1^IS^ range 0.313–10.258, with 5/6 patients with %BCR-ABL1^IS^ ≥1%.

## Discussion

Diagnostic assays for CML patients are based on standard testing using conventional cytogenetics, FISH and RQ-PCR. All three specifically detect the Philadelphia chromosome or *BCR-ABL1* fusion transcript [15]. Thanks to the introduction of targeted TKI therapy the majority of patients achieve cytogenetic and molecular remission, with a prolonged chronic phase and improved outcome. Thus, specific assays that monitor levels of *BCR-ABL1* are fundamental not only at diagnosis, but also for optimal patient management during therapy [8].

Recent evidence supports the diagnostic utility in employing a FC assay which captures and detects the presence of fusion proteins in leukemic cell lysates, such as the BCR-ABL1 protein in ALL [11] and PML-RARα protein in promyelocytic leukemia [16], where a rapid diagnosis is essential for patient management and treatment outcome [14]. For example, Raponi et al [17] applied the BCR-ABL1 protein immunoassay to ALL testing; the results were concordant with those obtained by conventional molecular techniques. On the basis of the above considerations, in the present study we tested a commercially available FCBA kit designed to detect the BCR-ABL1 fusion protein on primary CML samples in order to determine the applicability, reliability, specificity and rapidity of this method for both diagnosis and monitoring of CML patient burden within the initial months of therapy. After having standardized the FCBA method for our laboratory, we demonstrated that i) a minimum number of fresh collected cells are needed to avoid false negative results, ii) overlapping results can be achieved using either PB or BM cell samples, iii) all CML patients were properly diagnosed at the onset of the disease, iv) CML patients in follow-up showing detectable fusion protein (i.e. rMFI% >1) revealed levels of BCR-ABL1^IS^ transcripts between 1–10%, and v) rMFI% levels predict CCyR as defined by FISH analysis. Overall these results support the hypothesis that a chronic myeloproliferative disease clearly benefits from the FCBA approach for a rapid detection of BCR-ABL1 protein and diagnosis of t(9;22) CML, particularly in diagnostic laboratories where molecular testing facilities are either not available or have not been accredited and standardized according to the international guidelines, but are equipped with a flow-cytometer, as is the case for most hematology laboratories. More sensitive RQ-PCR analysis could be reserved, above all for the confirmation of diagnosis at onset and identification of patient specific BCR-ABL1 transcripts according to current recommendations, but more importantly be dedicated to the detection of MRD, following TKI treatment soon after FCBA negativity, which in turn may also surrogate FISH analysis.

Given the established efficacy of TKI treatment in CML, there is a compelling need for accurate methods able to monitor patient response or MRD at levels below the landmark of CCyR, which involves detection and quantification using the more sensitive RQ-PCR of BCR-ABL1 RNA, an excellent surrogate marker for long-term prognosis [18–20]. In patients treated with imatinib, a 3-log reduction (at least) in BCR-ABL1 transcripts from a standardized baseline value is associated with improved probability of long-term response and improved PFS. This desired value is considered the major molecular response (MMR) [21]. Patients who achieved CCyR and MMR after 18 months of imatinib therapy have an estimated 100% rate of PFS for 5 years [21].

In contrast to those patients where a steady decline in BCR-ABL1 transcripts indicates an ideal response to therapy [22], CML patients with a BCR-ABL1 value >10% at 3 months of therapy is statistically associated with poorer outcome, although many of these patients still achieve satisfactory outcomes [23–24]. Moreover, Bradford et al [25] recently showed the *rate* of BCR-ABL1 decline may be a critical prognostic discriminator of patients with very poor outcome among those >10% at 3 months, For the above reasons, patient monitoring using the FCBA protein rMFI% value as a semi-quantitative, indicator of patient response, since those patients with rMFI% value >1 after 3 months of TKI therapy could be flagged for the clinician who would then evaluate whether switching therapy to a newer generation TKI would be of benefit to the patient.

On the other hand, therapy suspension is currently a hot topic in CML circles since recent studies have shown that TKI therapy yields durable responses and prolongs survival. Yet, monitoring becomes even more relevant in special circumstances where the clinician may decide, along with the patient, to interrupt TKI therapy altogether for other reasons, for example, in patients who require therapy interruption due to pregnancy or adverse effects [26–27]. Although, more sensitive methods than conventional RQ-PCR have been used to detect residual disease in patients with undetectable BCR-ABL1, such as DNA PCRhttp://asheducationbook.hematologylibrary.org/content/2012/1/105.full-ref-7 [28], nanofluidic digital PCR [29], and replicate RQ-PCR [30], these methods are costly and not commonly available to all laboratories for routine use. This places the laboratory monitoring of CML patients in a critical position of rapidly identifying patients who may eventually relapse (i.e. with BCR-ABL1^IS^ transcripts above the MMR threshold 0.1%) [31–33] and/or rapidly progress to more advanced stages. In this context, the clinical laboratory requires a sensitive technique that is economical both in terms of time and costs both for CML screening and MRD follow-up. Most patients suspected of CML will be negative and this translates into avoiding subsequent, more costly molecular testing (including nested PCR and RQ-PCR) or samples being sent to accredited laboratories harmonized according to the International Scale. Despite many years of working toward international harmonization of BCR-ABL1 assessment, it has proved to be a complex process and is currently unavailable for many laboratories [34–35]. For patients who are positive a rapid and reliable screening test will result in earlier diagnosis and clinical intervention with TKIs.

Our data confirm those of previous studies indicating that the FCBA assay is sufficiently reliable as a first-line “screening” test for the patients suspected of CML using either PB or BM samples [36], thus reserving more invasive and costly techniques for confirmation of diagnosis and evaluation of cytogenetic abnormalities and atypical transcripts, in line with current CML guidelines. This is not a trivial concern considering the invasive nature of BM aspiration. In addition, we also show that for CML patients being monitored for MRD, applying this test is a simple and effective approach for the rapid screening of responses during the initial 3 or 6 months of TKI therapy to detect an early molecular response (EMR, defined as BCR-ABL1^IS^<10%) [37–38]. Then quantitative molecular tests from the peripheral blood samples alone may be sufficient to detect a BCR-ABL1^IS^ ratio below 1 − 10% on the International scale according to ELN 2013 Guidelines.

Our test cut-off predicted a quantitative BCR-ABL1^IS^ of approximately 1.6%. This indicates that the FCBA test can serve as a flag for poor response to therapy and/or drug failure, as an indication for switching TKIs, particularly to monitor a suspected progression of CML. This is particularly relevant considering the depth of the response obtained with TKI therapy, and the time to achieve this response (<1 year) are both important for the prediction of prognosis in the patient with CML. In fact, early response at 3 months of TKI treatment has become an important tool to predict favorable outcome [37–38]. About 70% of patients, those with fast initial response (BCR-ABL1^IS^ <10% at 3 months), face a 5-year overall survival (OS) of 95% [39], however a subgroup of patients experience progression to accelerated phase or blast crisis, predominantly in the first 3 years of treatment [40]. Therefore, in this context, a further advantage of the FCBA assay is that it can be used for monitoring rapid responses and predicting prognosis, offering the clinician the opportunity to request the test at more frequent intervals, monthly for instance in higher risk patients [41–43]. In addition, the rapidity of the FCBA method, provides the clinician with a lab result the same day as sampling, which is particularly important when a patient is suspected to be in progression or non-compliant with therapy.

CML patients are monitored by both cytogenetic and molecular assessments, with guidelines indicating to favor the switch from cytogenetic to molecular criteria. Lauseker et al [44] identified the BCR-ABL1^IS^ transcript level that can act as an equivalent substitute for CCyR, that is, the absence of detectable t(9;21) chromosomes [45]. The study suggests that, although there is no one-to-one cut-off for BCR-ABL1^IS^ representing CCyR, the cut-off of 1% BCR-ABL1^IS^ may be used to classify CCyR patients. In line with these data, the FCBA method can also substitute conventional cytogenetics or FISH in laboratories not equipped for these techniques for defining patients in CCyR if one expects the patient to have BCR-ABL1^IS^ transcript levels below 1%. Our results show that the FCBA can be at least as sensitive as FISH in monitoring patients for cytogenetic responses, it follows that when the FCBA assay is unable to detect BCR-ABL1 protein, further FISH testing is no longer indicated since results are highly likely to be negative, and only more sensitive molecular RQ-PCR follow-up is required for MRD. Instead, a positive FCBA assay is a strong indication for RQ-PCR testing which can be requested in a timely manner and in function of the relative MFI values obtained, since even in our relatively small sample size, appears to be proportional to the amount of protein detected and to the levels of transcript present.

We conclude that the BCR-ABL1 FCBA is an easy technique which detects BCR-ABL1 proteins translated from the more frequently occurring transcript types with sufficient specificity and sensitivity that can be easily integrated in the routine diagnosis of CML patients in clinical hematology laboratories equipped with a flow-cytometer. Moreover, the FCBA can be considered an additional tool for monitoring patient responses to TKI within the initial months of therapy, even at monthly intervals when BCR-ABL1 levels need to be monitored more frequently, particularly in those patients who are undergoing therapy suspension. In our laboratory setting, the assay showed detectable fusion protein corresponding to BCR-ABL1^IS^ transcripts around 1.6%, very close to the favorable CCyR response, with an improved detection over FISH. Certainly, substitution of FISH in favor of FCBA, and in MRD evaluation of transcripts, warrants testing in larger numbers of patients before a definitive conclusion can be made, although our data show that this assay may serve as a substitute for this technique in situations where the test is unavailable or fails.

However, as with any antibody based approach the specificity of the test depends entirely in the specificity of epitope of the antibody used for the BCR-ABL fusion protein. Hence, any eventual changes in the protein sequence of the epitope evolving over time (e.g. a partial deletion, the presence of a missense SNV, an acquired mutation) that could interfere with the recognition of the fusion protein by the antibody and will likely lead to a false negative. However, the chances of this occurring might be quite remote, yet this theoretical disadvantage is virtually existent in the FISH or RQ-PCR.

Undeniably, RQ-PCR currently and likely for many years will remain the gold standard for MRD evaluation in CML. However, the utility of the FCBA assay should not be ignored, the reliability and accuracy of the assay needs to be confirmed with larger numbers of patients and follow-up samples. Overall, sample detection is easy, rapid and sensitive and fully translatable to routine management of CML patients and a further advantage is that it is easily tailored to the needs of individual patients and of their clinicians. In the long run, savings in costs in terms of patient monitoring (approximately 40% savings in kits alone) of this methodology should provide substantial economic benefits when compared to FISH and RQ-PCR monitoring.

## Supporting Information

S1 Table Samples Tested Using the Flow Cytometric Immunobead Assay. (DOCX)

S1 Fig Effects of Thawing Cryopreserved Samples for the Detection of BCR-ABL1 Protein. Representative examples of change in fluorescence (MFI) obtained by flow-cyotmetry bead assay (FCBA) BCR-ABL test (A) using cell lines positive for BCR-ABL protein, and (B) CML patient total leukocytes at different time points. All samples at To (fresh lysates) tested positive. Tests were performed using fresh protein lysates and/or after thawing cells at different time points. All protein lysates were prepared according to the manufacturer’s protocol (see Materials and Methods). Negative FCBA-MFI Normal_cut off_ = 105 for this series.
(TIF)

S2 Fig Stability of peripheral blood samples at room temperature. (A) Peripheral blood samples were stored at room temperature for several days and then evaluated for BCR-ABL protein detection and cell viability using the flow-cyotmetry bead array (FCBA) BCR-ABL test and the 7-AAD assay, respectively. Data analysis was performed using Infinicyte software v1.0, BD Biosciences. Shown is a sample from an unusually stable patient (CML-05) (B) Time course comparison BCR-ABL Tests using SVP and BM samples from 2 different patients (CML-06 and CML-07). Negative FCBA-MFI Normal_cut off_ = 105 for this series.
(TIF)
