# Supplementary material for: Flow Cytometric Immunobead Assay for Detection of BCR-ABL1 Fusion Proteins in Chronic Myleoid Leukemia: Comparison with FISH and PCR Techniques
Source: PLoS One. 2015 Jun 25;10(6):e0130360. doi: 10.1371/journal.pone.0130360 (PMC4482505; doi:10.1371/journal.pone.0130360)
Supplement: S1 Table — (DOCX) [file pone.0130360.s001.docx]

**Supplementary Table 1. Samples tested using the flow cytometric immunobead assay (FCBA)**

| Samples Tested | #Samples (BM/PB) | #Patients | Male/Female ratio |
| --- | --- | --- | --- |
| *CML* |  |  |  |
| Diagnosis* | 88 (35/53) | 63 | 45/18 |
| Follow-Up** | 153 (50/103) | 55 | 29/26 |
| B-Lineage ALL | 14 (10/4) | 13 | 6/7 |
| AML | 4 (1/3) | 4 | 0/4 |
| Other*** | 19 (5/14) | 14 | 8/7 |
| Total | 278 | 122 |  |

FCBA= Flow-cytometry Bead Assay

Samples tested (N=278); Patients tested (N=122)

*N=28 patients tested in both follow-up and diagnosis;

**Patients tested in follow-up with mean 1.8 tests/pt (range 1-18 samples tested, samples from 3months to 36 months);

BM= bone marrow, PB= peripheral blood

***Including (samples/patient): AML (4/4), other hematological malignancies

[lymphoma (3/2), CMML (4/3); MPN (TE, PV, MFI, 12/9)].
