# Supplementary figures and images for: Flow Cytometric Immunobead Assay for Detection of BCR-ABL1 Fusion Proteins in Chronic Myleoid Leukemia: Comparison with FISH and PCR Techniques
Source: PLoS One. 2015 Jun 25;10(6):e0130360. doi: 10.1371/journal.pone.0130360 (PMC4482505; doi:10.1371/journal.pone.0130360)

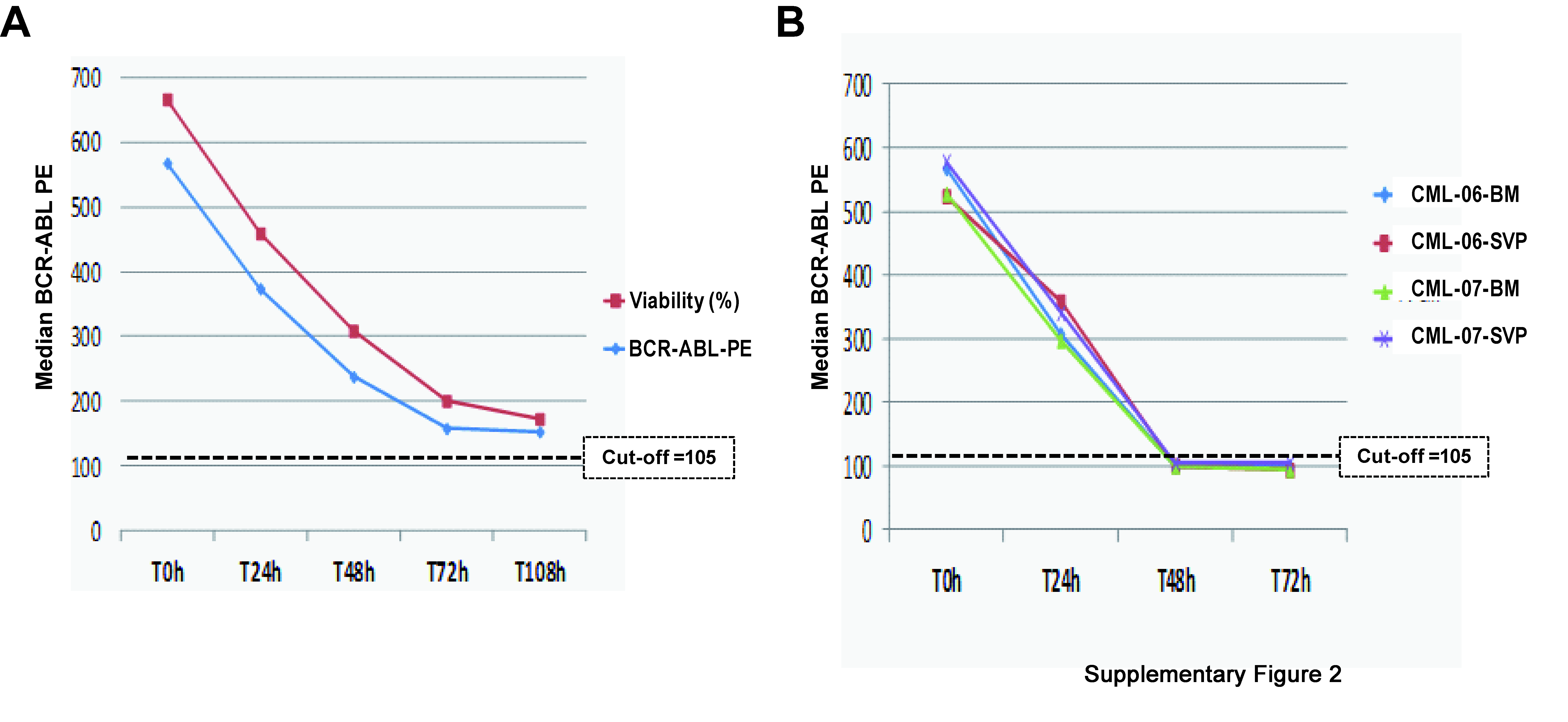

Supplement: S2 Fig — (A) Peripheral blood samples were stored at room temperature for several days and then evaluated for BCR-ABL protein detection and cell viability using the flow-cyotmetry bead array (FCBA) BCR-ABL test and the 7-AAD assay, respectively. Data analysis was performed using Infinicyte software v1.0, BD Biosciences. Shown is a sample from an unusually stable patient (CML-05) (B) Time course comparison BCR-ABL Tests using SVP and BM samples from 2 different patients (CML-06 and CML-07). Negative FCBA-MFI Normalcut off = 105 for this series. (TIF) [file pone.0130360.s003.tif]
